# Association of Cannabis Use During Adolescence With Neurodevelopment

**DOI:** 10.1001/jamapsychiatry.2021.1258

**Published:** 2021-06-16

**Authors:** Matthew D. Albaugh, Jonatan Ottino-Gonzalez, Amanda Sidwell, Claude Lepage, Anthony Juliano, Max M. Owens, Bader Chaarani, Philip Spechler, Nicholas Fontaine, Pierre Rioux, Lindsay Lewis, Seun Jeon, Alan Evans, Deepak D’Souza, Rajiv Radhakrishnan, Tobias Banaschewski, Arun L. W. Bokde, Erin Burke Quinlan, Patricia Conrod, Sylvane Desrivières, Herta Flor, Antoine Grigis, Penny Gowland, Andreas Heinz, Bernd Ittermann, Jean-Luc Martinot, Marie-Laure Paillère Martinot, Frauke Nees, Dimitri Papadopoulos Orfanos, Tomáš Paus, Luise Poustka, Sabina Millenet, Juliane H. Fröhner, Michael N. Smolka, Henrik Walter, Robert Whelan, Gunter Schumann, Alexandra Potter, Hugh Garavan

**Affiliations:** 1 Department of Psychiatry, University of Vermont Larner College of Medicine, Burlington; 2 McConnell Brain Imaging Centre, McGill University, Montreal, Quebec, Canada; 3 Department of Psychiatry, Yale University School of Medicine, New Haven, Connecticut; 4 Department of Child and Adolescent Psychiatry and Psychotherapy, Central Institute of Mental Health, Medical Faculty Mannheim, Heidelberg University, Mannheim, Germany; 5 Discipline of Psychiatry, School of Medicine and Trinity College Institute of Neuroscience, Trinity College Dublin, Dublin, Ireland; 6 Centre for Population Neuroscience and Precision Medicine, Institute of Psychiatry, Psychology, and Neuroscience, Social, Genetic & Developmental Psychiatry Centre, King’s College London, London, United Kingdom; 7 Department of Psychiatry, University of Montreal, Montreal, Quebec, Canada; 8 Institute of Cognitive and Clinical Neuroscience, Central Institute of Mental Health, Medical Faculty Mannheim, Heidelberg University, Mannheim, Germany; 9 Department of Psychology, School of Social Sciences, University of Mannheim, Mannheim, Germany; 10 NeuroSpin, Commissariat à l’Energie Atomique, Université Paris-Saclay, Gif-sur-Yvette, France; 11 Sir Peter Mansfield Imaging Centre School of Physics and Astronomy, University of Nottingham, University Park, Nottingham, United Kingdom; 12 Department of Psychiatry and Psychotherapy Campus Charité Mitte, Charité–Universitätsmedizin Berlin, Berlin, Germany; 13 corporate member of Freie Universität Berlin, Humboldt-Universität zu Berlin, Berlin, Germany; 14 Berlin Institute of Health, Berlin, Germany; 15 Physikalisch-Technische Bundesanstalt, Berlin, Germany; 16 Institut National de la Santé et de la Recherche Médicale U A10 “Trajectoires développementales en psychiatrie” Université Paris-Saclay, Ecole Normale supérieure Paris-Saclay, CNRS, Centre Borelli, Gif-sur-Yvette, France; 17 Institut National de la Santé et de la Recherche Médicale, INSERM U A10 “Trajectoires développementales en psychiatrie,” Paris, France; 18 Université Paris-Saclay, Ecole Normale supérieure Paris-Saclay, CNRS, Centre Borelli, Paris, France; 19 AP-HP Sorbonne Université, Department of Child and Adolescent Psychiatry, Pitié-Salpêtrière Hospital, Paris, France; 20 Institute of Medical Psychology and Medical Sociology, University Medical Center Schleswig Holstein, Kiel University, Kiel, Germany; 21 Bloorview Research Institute, Holland Bloorview Kids Rehabilitation Hospital, Toronto, Ontario, Canada; 22 Department of Psychology, University of Toronto, Toronto, Ontario, Canada; 23 Department of Psychiatry, University of Toronto, Toronto, Ontario, Canada; 24 Department of Child and Adolescent Psychiatry and Psychotherapy, University Medical Centre Göttingen, Göttingen, Germany; 25 Department of Psychiatry and Neuroimaging Center, Technische Universität Dresden, Dresden, Germany; 26 School of Psychology and Global Brain Health Institute, Trinity College Dublin, Ireland; 27 Centre for Population Neuroscience and Precision Medicine, Institute of Psychiatry, Psychology, and Neuroscience, Social, Genetic & Developmental Psychiatry Centre, King’s College London, London, United Kingdom; 28 Centre for Population Neuroscience and Precision Medicine Research Group, Department of Psychiatry and Psychotherapy, Campus Charite Mitte, Humboldt University, Berlin, Germany; 29 Leibniz Institute for Neurobiology, Magdeburg, Germany; 30 Institute for Science and Technology of Brain-inspired Intelligence, Fudan University, Shanghai, PR China

## Abstract

**Importance:**

Animal studies have shown that the adolescent brain is sensitive to disruptions in endocannabinoid signaling, resulting in altered neurodevelopment and lasting behavioral effects. However, few studies have investigated ties between cannabis use and adolescent brain development in humans.

**Objective:**

To examine the degree to which magnetic resonance (MR) imaging–assessed cerebral cortical thickness development is associated with cannabis use in a longitudinal sample of adolescents.

**Design, Setting, and Participants:**

Data were obtained from the community-based IMAGEN cohort study, conducted across 8 European sites. Baseline data used in the present study were acquired from March 1, 2008, to December 31, 2011, and follow-up data were acquired from January 1, 2013, to December 31, 2016. A total of 799 IMAGEN participants were identified who reported being cannabis naive at study baseline and had behavioral and neuroimaging data available at baseline and 5-year follow-up. Statistical analysis was performed from October 1, 2019, to August 31, 2020.

**Main Outcomes and Measures:**

Cannabis use was assessed at baseline and 5-year follow-up with the European School Survey Project on Alcohol and Other Drugs. Anatomical MR images were acquired with a 3-dimensional T1-weighted magnetization prepared gradient echo sequence. Quality-controlled native MR images were processed through the CIVET pipeline, version 2.1.0.

**Results:**

The study evaluated 1598 MR images from 799 participants (450 female participants [56.3%]; mean [SD] age, 14.4 [0.4] years at baseline and 19.0 [0.7] years at follow-up). At 5-year follow-up, cannabis use (from 0 to >40 uses) was negatively associated with thickness in left prefrontal (peak: *t*_785_ = –4.87, cluster size = 1558 vertices; *P* = 1.10 × 10^−6^, random field theory cluster corrected) and right prefrontal (peak: *t*_785_ = –4.27, cluster size = 1551 vertices; *P* = 2.81 × 10^−5^, random field theory cluster corrected) cortices. There were no significant associations between lifetime cannabis use at 5-year follow-up and baseline cortical thickness, suggesting that the observed neuroanatomical differences did not precede initiation of cannabis use. Longitudinal analysis revealed that age-related cortical thinning was qualified by cannabis use in a dose-dependent fashion such that greater use, from baseline to follow-up, was associated with increased thinning in left prefrontal (peak: *t*_815.27_ = –4.24, cluster size = 3643 vertices; *P* = 2.28 × 10^−8^, random field theory cluster corrected) and right prefrontal (peak: *t*_813.30_ = –4.71, cluster size = 2675 vertices; *P* = 3.72 × 10^−8^, random field theory cluster corrected) cortices. The spatial pattern of cannabis-related thinning was associated with age-related thinning in this sample (*r* = 0.540; *P* < .001), and a positron emission tomography–assessed cannabinoid 1 receptor–binding map derived from a separate sample of participants (*r* = −0.189; *P* < .001). Analysis revealed that thinning in right prefrontal cortices, from baseline to follow-up, was associated with attentional impulsiveness at follow-up.

**Conclusions and Relevance:**

Results suggest that cannabis use during adolescence is associated with altered neurodevelopment, particularly in cortices rich in cannabinoid 1 receptors and undergoing the greatest age-related thickness change in middle to late adolescence.

## Key Points

**Question:**

To what extent is cannabis use associated with magnetic resonance imaging–measured cerebral cortical thickness development during adolescence?

**Findings:**

In this cohort study, linear mixed-effects model analysis using 1598 magnetic resonance images from 799 participants revealed that cannabis use was associated with accelerated age-related cortical thinning from 14 to 19 years of age in predominantly prefrontal regions. The spatial pattern of cannabis-related cortical thinning was significantly associated with a positron emission tomography–assessed map of cannabinoid 1 receptor availability.

**Meaning:**

Results suggest that cannabis use during middle to late adolescence may be associated with altered cerebral cortical development, particularly in regions rich in cannabinoid 1 receptors.

## Teaser

This cohort study examines the degree to which magnetic resonance imaging–assessed cerebral cortical thickness development is associated with cannabis use in a longitudinal sample of adolescents.

## Introduction

Cannabis is a commonly used psychoactive drug, particularly among adolescents and young adults. Relative to the general population, past-year prevalence rates of cannabis use are greatest among teenagers, and more than one-third of 12th graders in the United States report using cannabis in the past year.^1,2^ Seventy-eight percent of first-time cannabis users are between the ages of 12 and 20 years.^3^ These prevalence rates raise concern as cannabis use during adolescence has been linked to enduring impairments of executive functioning and impulse control.^4^ Such longitudinal associations appear specific to cannabis use and independent of concomitant alcohol use; however, the neurobiological mechanisms that might mediate a long-term behavioral association with cannabis use remain unclear.^4^ The potential association of cannabis use with adolescent development represents an increasingly relevant public health issue, particularly given evidence of increased problematic cannabis use among adolescents in areas where recreational cannabis use has been legalized.^5^

The transition from late adolescence to early adulthood is characterized by significant structural change in the brain, most notably in areas of the cerebral cortex that are known to exhibit protracted developmental trajectories and undergo relatively late myelination.^6,7,8,9^ Extant research studies suggest that changes in endocannabinoid signaling can have a significant association with aspects of mammalian brain development.^10,11^ Evidence further indicates that the adolescent brain may be particularly sensitive to disruptions in normative fluctuations in endocannabinoid signaling, associated with altered neurodevelopment and behavior.^12,13,14,15^ Despite such findings in the animal literature, few longitudinal neuroimaging studies have examined putative ties between cannabis use and adolescent brain development, to our knowledge.

Here, we examined the association between cannabis use and cerebral cortical development in a longitudinal, community-based sample of adolescents. From the larger IMAGEN sample, we identified participants who reported being cannabis naive at study baseline and had neuroimaging data available at study baseline and 5-year follow-up. First, in a series of cross-sectional analyses, we examined the extent to which lifetime cannabis use was associated with cortical thickness at 5-year follow-up (approximately 19 years of age). To test the temporality of this association, we then examined the extent to which cortical thickness at age 14 years was associated with lifetime cannabis use at 5-year follow-up. In our primary longitudinal analysis, a linear mixed-effects model (LMM) was implemented to test the degree to which initiation of cannabis use was associated with age-related cortical thickness change (from ages 14 to 19 years). Follow-up analyses were conducted to test the extent to which cannabis-related cortical thinning was associated with aspects of impulsive behavior. We also tested the association between the longitudinally derived map of cannabis-related cortical thinning and positron emission tomography (PET)–derived cannabinoid 1 (CB1) receptor availability (collected from an independent sample of young adults) with the hypothesis that areas demonstrating cannabis-related thinning would exhibit, on average, relatively greater CB1 receptor availability. We further hypothesized that cannabis-related thinning would be most evident in cortical regions undergoing the greatest structural change during the developmental window studied.

## Methods

### Sample

Neuroimaging and behavioral data were obtained from the IMAGEN study,^16^ conducted across 8 European sites, which includes 2223 adolescents recruited from schools at approximately 14 years of age (range, 12.9-15.7 years). Baseline data used in the present cohort study were acquired from March 1, 2008, to December 31, 2011, and follow-up data were acquired from January 1, 2013, to December 31, 2016. Local ethics research committees approved the study at each site (London, England: Psychiatry, Nursing and Midwifery Research Ethics Subcommittee, Waterloo Campus, King’s College London; Nottingham, England: University of Nottingham Medical School Ethics Committee; Mannheim, Germany: Medizinische Fakultaet Mannheim, Ruprecht Karl Universitaet Heidelberg and Ethik-Kommission II an der Fakultaet fuer Kliniksche Medizin Mannheim; Dresden, Germany: Ethikkommission der Medizinischen Fakultaet Carl Gustav Carus, TU Dresden Medizinische Fakultaet; Hamburg, Germany: Ethics Board, Hamburg Chamber of Physicians; Paris, France: CPP IDF VII (Comité de protection des personnes Ile de France), ID RCB: 2007-A00778-45 September 24, 2007; Dublin, Ireland: TCD School of Psychology REC; and Berlin, Germany: Ethics Committee of the Faculty of Psychology). Written consent was obtained from the adolescent’s parent or guardian, and verbal assent was obtained from the adolescent. We identified 799 participants who reported being cannabis naive on the European School Survey Project on Alcohol and Other Drugs (ESPAD)^17^ at study baseline and had behavioral and quality-controlled neuroimaging data available at study baseline and 5-year follow-up.

### Substance Use Measures

Substance use was assessed at baseline and 5-year follow-up with ESPAD,^17^ a self-report questionnaire that measures use of alcohol, nicotine, and cannabis as well as other substances. Participants indicated how frequently they had used each of the substances in their lifetime, in the past 12 months, in the past 30 days, and in the past 7 days using a 7-point scale (where 0 indicates never; 1, 1-2 times; 2, 3-5 times; 3, 6-9 times; 4, 10-19 times; 5, 20-39 times; and 6, ≥40 times).

The Alcohol Use Disorders Identification Test (AUDIT) is a 10-item screening tool created by the World Health Organization that assesses alcohol consumption, drinking behaviors, and alcohol-associated problems.^18^ AUDIT was administered to youths at baseline and follow-up. The AUDIT Alcohol Consumption scale (AUDIT-C) was used in the present study and is composed of items on AUDIT that explicitly assess the amount and frequency of alcohol consumption.^19,20^

### Impulsivity Measures

Given prior research suggesting that cannabis use has associations with impulse control, we chose to examine associations between cannabis-related thinning and 3 domains of impulsiveness (attentional, nonplanning, and motor) assessed on the Barratt Impulsiveness Scale,^21,22^ a 30-item self-report questionnaire that was administered at 5-year follow-up in IMAGEN.

### Cortical Thickness

Anatomical magnetic resonance (MR) images were acquired with a 3-dimensional T1-weighted magnetization prepared gradient echo sequence based on the Alzheimer’s Disease Neuroimaging Initiative protocol.^23^ Quality-controlled native MR images were processed through the CIVET pipeline, version 2.1.0 (Montreal Neurological Institute) using the CBRAIN platform (Montreal Neurological Institute)^24^ and Compute Canada^25^ (eAppendix 1 in the Supplement).

### CB1 Receptor Availability

To test for possible associations between the spatial distribution of cannabis-related cortical thinning and a receptor for the endocannabinoid system, we used a map of CB1 receptor availability generated from healthy control participants in a previously published study.^26^ Maps of CB1 receptor availability were generated using PET and the reversible ligand [^11^C]OMAR in 21 men aged 18 to 35 years. The 21 individual participant maps were averaged to provide an estimate of CB1 receptor availability at each voxel. This mean PET volume was subsequently projected to a cortical surface model in the Montreal Neurological Institute International Consortium for Brain Mapping space.

### Statistical Analysis

Statistical analysis was performed from October 1, 2019, to August 31, 2020. Cortical thickness analysis was implemented using SurfStat, a toolbox created for MATLAB (The MathWorks Inc).^27^ In cross-sectional analyses, local cortical thickness was regressed on lifetime cannabis use. Longitudinal cortical thickness analysis was conducted using LMMs.^8,28,29,30,31,32,33^ In LMMs, participant ID was entered as a random effect to account for within-individual dependence. Change in lifetime cannabis use (from baseline to 5-year follow-up) was included as a time-invariant covariate. Age, total brain volume, sex, handedness, site, and consumption score on AUDIT were controlled for in all analyses. To account for multiple comparisons, random field theory correction was applied to the cortical surface (eAppendix 2 in the Supplement).^34^ A random field theory cluster–corrected significance threshold of *P* < .05 was used for all cortical thickness analyses.

## Results

### Demographics and Cannabis Use

The study evaluated 1598 MR images from 799 participants (450 female participants [56.3%]; mean [SD] age at baseline, 14.4 [0.4] years). Demographic information is summarized in the Table and eTable 1 in the Supplement. Demographic information regarding excluded IMAGEN participants can be found in eTable 2 in the Supplement. At follow-up, lifetime cannabis use ranged from 0 to more than 40 uses, with 208 participants reporting 1 to 9 uses and 161 participants reporting 10 to more than 40 uses. Distribution of lifetime cannabis use at 5-year follow-up is shown in eFigure 1 in the Supplement. Descriptive statistics are provided for ESPAD substance use items and AUDIT-C in eTables 3-6 in the Supplement. For further details regarding demographic variables, see eAppendix 3 in the Supplement.

**Table.** Summary Statistics for Demographic Variables

Characteristic	Total, mean (SD) (N = 799)
Age, y	
Baseline	14.4 (0.4)
Follow-up	19.0 (0.7)
Sex, No. (%)	
Female	450 (56.3)
Male	349 (43.7)
Baseline	
Socioeconomic status^a^	18.2 (3.7)
Verbal IQ	112.6 (13.0)
Performance IQ	109.6 (13.6)

^a^ Details for the socioeconomic score can be found in eAppendix 1 of the Supplement.

### Cannabis Use and Cortical Thickness

#### Cross-Sectional

**Figure 1.**
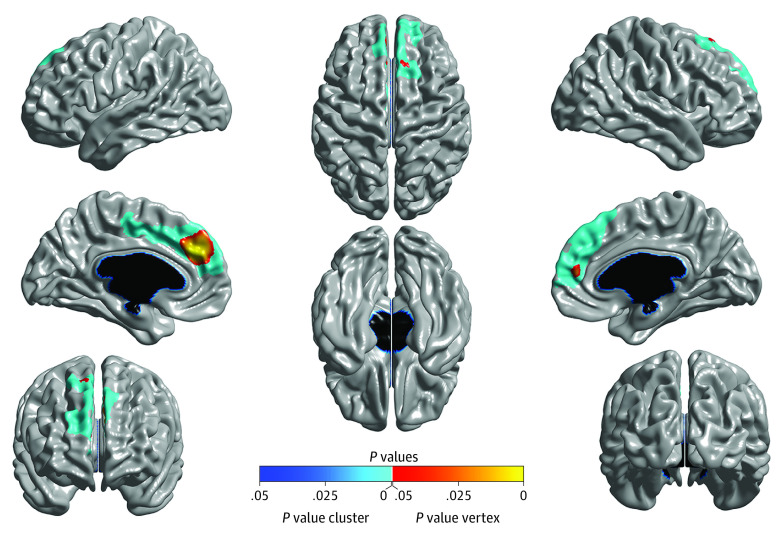
Cross-Sectional Results Brain areas where local cortical thickness is negatively associated with the dimensional measure of lifetime cannabis use at 5-year follow-up (N = 799). Random field theory was used to correct for multiple comparisons over the entire cortical mantle. The figure is shown at *P* ≤ .05, random field theory corrected. Blue areas are significant at the cluster level, and red corresponds to areas significant at the vertex level. Measures were controlled for age, total brain volume, sex, handedness, Alcohol Use Disorders Identification Test Alcohol Consumption score, and site.

At 5-year follow-up, there was evidence of a dose-dependent association between lifetime cannabis use and cortical thickness (n = 799), with significant negative associations between lifetime cannabis use and thickness in left prefrontal (peak: *t*_785_ = –4.87, cluster size = 1558 vertices; *P* = 1.10 × 10^−6^, random field theory cluster corrected) and right prefrontal (peak: *t*_785_ = –4.27, cluster size = 1551 vertices; *P* = 2.81 × 10^−5^, random field theory cluster corrected) cortices (Figure 1). There were no significant associations between baseline cortical thickness and follow-up lifetime cannabis use, suggesting that the neuroanatomical differences observed at 5-year follow-up did not precede initiation of cannabis use. Even when reducing the statistical threshold to *P* ≤ .005 uncorrected, only several negative associations were revealed—and these areas were well outside of those showing the 5-year follow-up associations (eFigure 2 in the Supplement).

#### Longitudinal

**Figure 2.**
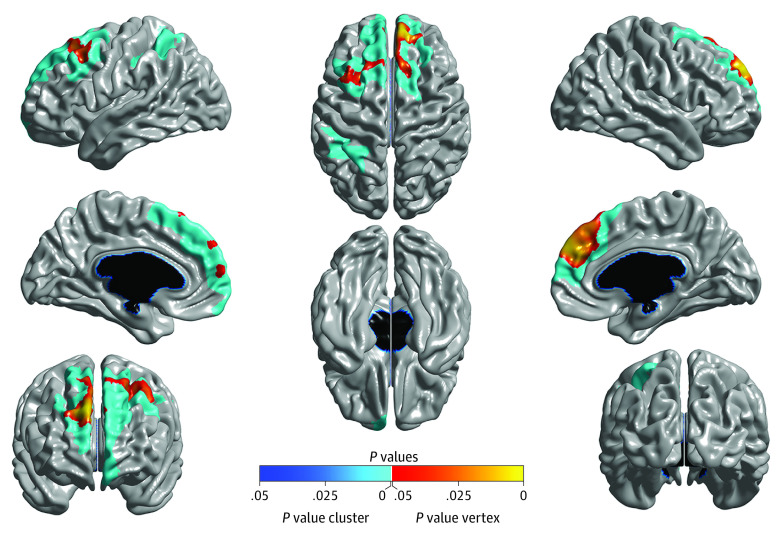
Longitudinal Linear Mixed-Effects Model Results Brain areas where local cortical thickness is associated with the time × cannabis interaction in a linear mixed-effects model analysis, controlling for the main effects of time point, lifetime cannabis use, total brain volume, sex, handedness, Alcohol Use Disorders Identification Test Alcohol Consumption score, and site (N = 799; 1598 magnetic resonance imaging scans). The figure is shown at *P* ≤ .05 with a whole-brain random field theory correction. Blue shades correspond to areas significant at the cluster level and red shades to areas significant at the vertex level.

**Figure 3.**
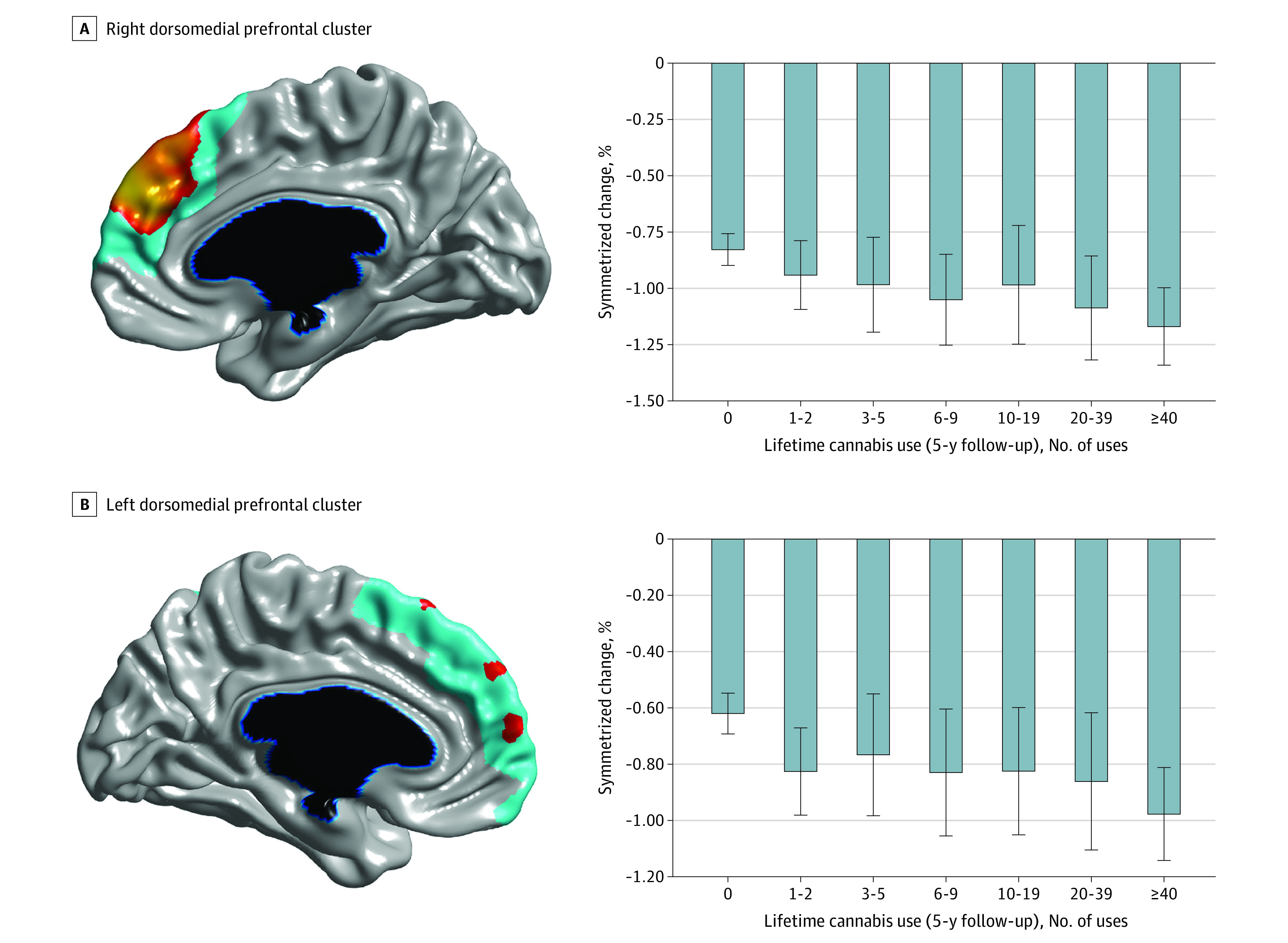
Magnetic Resonance Imaging–Assessed Cortical Thinning at Varying Levels of Lifetime Cannabis Use A, Right dorsomedial prefrontal cluster from linear mixed-effects analysis. B, Left dorsomedial prefrontal cluster from linear mixed-effects analysis. The bar graphs depict within-individual symmetrized percentage change (ie, change in cortical thickness, in millimeters per year, with respect to the mean cortical thickness across both time points) for each cluster at varying levels of lifetime cannabis use (at 5-year follow-up). Error bars represent 95% confidence intervals. Brain figures shown at *P* ≤ .05 with a whole-brain random field theory correction. Blue shades correspond to areas significant at the cluster level, and orange shades to areas significant at the vertex level.

**Figure 4.**
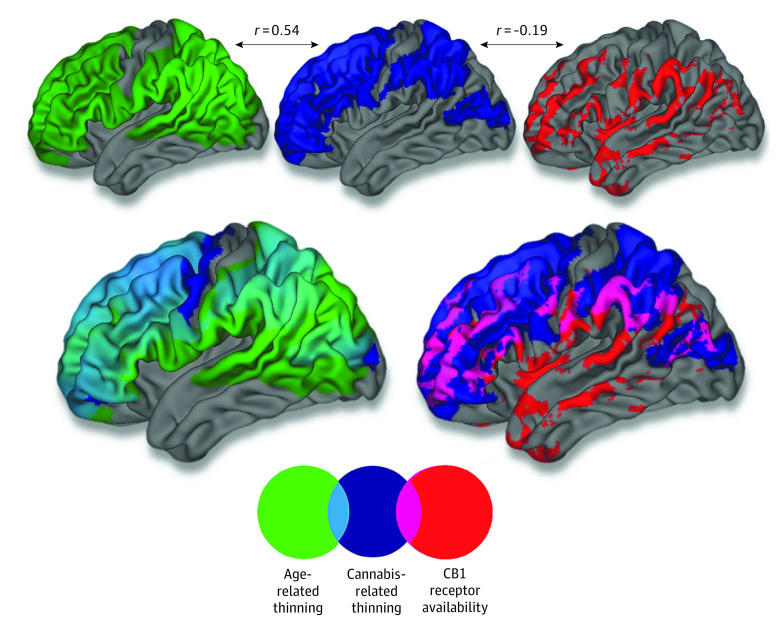
Topographical Overlap Between Age-Related Thinning, Cannabis Effect, and Cannabinoid 1 (CB1) Receptor Availability Topographical overlap between age-related cortical thinning in the sample (n = 799), areas in which age-related thinning was qualified by cannabis use, and positron emission tomography–assessed CB1 receptor availability (collected from a separate sample of 21 healthy adults). The *r* values correspond to Pearson correlation coefficients between unthresholded vertex-level surface maps. Please note that thresholds have been lowered for visualization purposes. Regional [^11^C]OMAR volume distribution is shown at >1.4, age-related thinning map is shown at *t* < −15, and cannabis-related thinning map is shown at *t* < −2.

In line with the cross-sectional results, longitudinal LMM analysis (799 participants and 1598 MR images) revealed a significant time × cannabis interaction such that cannabis use was associated with accelerated age-related cortical thinning in left prefrontal (peak: *t*_815.27_ = –4.24, cluster size = 3643 vertices; *P* = 2.28 × 10^−8^, random field theory cluster corrected) and right prefrontal (peak: *t*_813.30_ = –4.71, cluster size = 2675 vertices; *P* = 3.72 × 10^−8^, random field theory cluster corrected) cortices (Figure 2 and Figure 3; eFigure 3 in the Supplement). Results were not meaningfully altered when controlling for baseline age and length of time between study visits. Further, the unthresholded *t* statistic map for the time × cannabis interaction was significantly associated with a PET-derived map of CB1 receptor availability (collected on a separate sample of 21 healthy adults) (*r* = −0.189; *P* < .001), indicating that cortical areas in which age-related thinning was qualified by cannabis partially overlapped with areas showing a higher density of CB1 receptors as indexed by [^11^C]OMAR binding (Figure 4).

Given that PET data were collected on an all-male sample, we reran our LMM using male participants only (n = 349 and 698 MR images). The *t* map for the time × cannabis interaction in male participants was similar to results obtained when male and female participants were analyzed together. Furthermore, the unthresholded *t* statistic map for the time × cannabis interaction in male participants was significantly associated with the PET-derived map of CB1 receptor availability (*r* = −0.313; *P* < .001).

### Age and Cortical Thickness

Next, longitudinal LMM analysis was implemented to characterize the association between age and cortical thickness in the sample of 799 participants who were cannabis naive at baseline. Consistent with prior reports of cortical thickness development, there was a significant main association of time point with cortical thickness, with most areas of the cortex evidencing age-related thinning.^7,8^ The spatial pattern of cannabis-related cortical thinning was correlated with the unthresholded *t* statistic map for the association with time, indicating that, on average, cannabis-related thinning was greater in cortical regions evidencing the most significant age-related thinning in this sample (*r* = 0.540; *P* < .001) (Figure 4).

### Additional Covariates, Moderators, and Cannabis Use Variables

Across all analyses, controlling for socioeconomic status, verbal IQ, and performance IQ did not meaningfully alter results. In cross-sectional and longitudinal analyses, we examined sex as a potential moderator in the association between cortical thickness and cannabis use. In cross-sectional analyses, there was no significant sex × cannabis interaction on cortical thickness. Similarly, in longitudinal analysis, a time × cannabis × sex interaction was not significantly associated with cortical thickness, indicating that the association between age-related thinning and cannabis use did not statistically differ between sexes. Nearly identical results were obtained when all analyses were rerun using a binary cannabis use variable (moderate and heavy users vs cannabis naive) with a between-group design. See eAppendix 4, eFigure 4, and eFigure 5 in the Supplement for details. Although alcohol consumption was controlled for in the above analyses, co-occurring tobacco use represents an additional potential confounder. At 5-year follow-up, lifetime tobacco use was correlated with lifetime cannabis use on ESPAD (*r* = 0.573; *P* < .001). However, rerunning the longitudinal analysis and including lifetime tobacco use as a covariate resulted in largely consistent findings (eFigure 6 in the Supplement).

### Cannabis-Related Thinning and Impulsiveness

Cannabis-related cortical thinning in the right dorsomedial prefrontal cortex accounted for unique variance in attentional impulsiveness at 5-year follow-up while controlling for sex, site, baseline age, baseline brain volume, baseline pubertal development, verbal IQ, and performance IQ (*b* = −0.119; *P* = .003). Thus, accelerated thinning in the right dorsomedial prefrontal cortex was associated with the transition to cannabis use as well as greater attentional impulsiveness at 5-year follow-up. This association held even when controlling for baseline parent-reported and self-reported attention-deficit/hyperactivity disorder symptoms (eAppendix 5 in the Supplement). Exploratory follow-up analyses revealed no significant associations between cannabis-associated thinning and other psychopathologic and neurocognitive measures (eAppendix 6 and eAppendix 7 in the Supplement).

## Discussion

To our knowledge, the present investigation represents the largest longitudinal neuroimaging study of cannabis use to date. Results suggest that cannabis use during middle to late adolescence may be associated with altered cortical development, particularly in prefrontal regions rich in CB1 receptors and exhibiting protracted maturational trajectories. Specifically, we found evidence of a dose-dependent association between cannabis use from baseline to 5-year follow-up and accelerated cortical thinning during that same period, primarily in prefrontal regions. Baseline cortical thickness was not associated with lifetime cannabis use at 5-year follow-up, suggesting that the observed neuroanatomical associations with lifetime cannabis use were not associated with preexisting differences in brain structure. Results from longitudinal analysis indicated that age-related cortical thinning was associated with cannabis use in a dose-dependent fashion such that greater use from baseline to 5-year follow-up was associated with increased rates of cortical thinning in predominantly prefrontal regions during that same period. Our results are corroborated by convergence with PET mapping of CB1 receptor availability; cortical areas in which the transition to cannabis use was associated with accelerated age-related thinning were, on average, cortical regions with increased CB1 receptor availability. Across analyses, we controlled for co-occurring alcohol consumption and confirmed that the associations with cannabis use persisted when covarying for nicotine use. Follow-up analyses indicate a potential consequence of cannabis-related cortical thinning, as cortical thinning in the right dorsomedial prefrontal cortex from baseline to 5-year follow-up was associated with attentional impulsiveness at 5-year follow-up.

Numerous cross-sectional studies have tested for brain structural correlates of adolescent cannabis use, although findings have been inconsistent.^35,36,37,38,39,40,41,42^ In general, when comparing adolescent cannabis users with nonusers, cross-sectional studies have reported evidence of reduced volume and surface area across frontal and parietal areas as well as reduced cortical thickness in frontal regions.^35,38,43^ Other studies have found evidence of increased volume and/or thickness in temporal and cerebellar regions in adolescent cannabis users relative to peers who did not use cannabis.^37,41,42^ However, some prior studies have failed to reveal structural differences between adolescent cannabis users and controls who did not use cannabis.^39,40^ Few longitudinal neuroimaging studies have attempted to test for associations between change in cannabis use and change in brain structure. In a study of 30 adolescents with heavy marijuana use and concomitant alcohol use, Jacobus et al^44^ found evidence of attenuated age-related thinning in comparison with controls, predominantly in frontal and parietal regions such that greater cumulative marijuana use was associated with increased thickness estimates at 3-year follow-up. However, participants in this prior study ranged from 16 to 19 years of age at baseline, spanning a broad neurodevelopmental window. In a smaller sample of IMAGEN participants, French et al^45^ reported evidence of cortical thickness reductions associated with cannabis use; however, cannabis-related cortical thickness reductions were found in males only.

It has long been postulated that ongoing neurodevelopmental processes during adolescence may impart heightened vulnerability to cannabis exposure and increase the likelihood of long-term associations with cognition and behavior. Many animal studies have reported enduring effects of adolescent exposure to tetrahydrocannabinol (THC), the primary psychoactive substance in cannabis. Specifically, adolescent exposure to THC has been shown to decrease social behavior in adult rats^46,47^ as well as alter motivational processes.^48^ Rodent and primate studies have also demonstrated that adolescent exposure to THC results in working memory deficits in adulthood.^49,50,51,52^ Several rodent studies have also found that adolescent THC exposure results in lasting alterations in glutamatergic and γ-aminobutyric acid–ergic functioning.^53,54^ In humans, adolescent-onset cannabis users exhibit greater use-associated problems in adulthood relative to late-onset cannabis users.^55,56^ Findings from the present study may help to elucidate heightened vulnerability to the effects of cannabis use among adolescents. We found that the statistical map of age-related cortical change was significantly correlated with statistical maps of the time × cannabis interaction on cortical thickness as well as the main association of cannabis use with cortical thickness at 5-year follow-up. Taken together, these results suggest that, on average, cannabis use tended to qualify cortical thickness change within areas already undergoing the greatest degree of age-related change (from baseline to 5-year follow-up). This finding provides support for the association of cannabis use with ongoing maturational processes in the brain and a possible explanation for the heightened vulnerability to the cognitive outcomes of cannabis use among adolescents. More important, our imaging findings are consistent with recent animal research on adolescent THC exposure and prefrontal cortical maturation. Miller et al^15^ examined the association of adolescent THC exposure with prefrontal cortical maturation using a rat model. Researchers injected male rats with THC during the period of their adolescence, spanning 4 to 7 weeks of age. They found that adolescent THC exposure resulted in distinct proximate and long-term alterations of dendritic architecture. Specifically, THC exposure disrupted normal neurodevelopmental processes by inducing premature pruning of dendritic spines and atrophy of dendritic arbors in early adulthood. We hypothesize that the MR imaging (MRI)–assessed cannabis-related thinning revealed in our human study is underpinned by the same neurobiological phenomenon.

### Strengths and Limitations

Our study possesses several strengths that may help to explain apparent discrepancies when comparing our findings with those of previous longitudinal imaging studies of cannabis use. First, all participants in the present study were reportedly cannabis naive at baseline, and, for those who transitioned to cannabis use, exposure occurred during the same developmental window—a critical detail given that the associations of cannabis exposure may be largely dependent on neurodevelopmental stage. Second, the number of participants in the present study offers increased statistical power to detect relatively subtle brain changes.

Several limitations of the present study should also be addressed. The PET data used in this study were collected on a separate sample of young adults, not the 799 youths who underwent longitudinal neuroimaging. Given the invasive nature of PET imaging and its associated risks, it is not ethical to collect PET data on minors. We cannot state definitively that, in our sample of 799 participants, the areas exhibiting cannabis-related thinning in longitudinal MRI analysis were, in fact, high in CB1 receptor availability. Our present findings are also limited by the self-report nature of our cannabis use measure. As with any self-report measure, it is possible that participants were not honest regarding their cannabis use or that their estimates of past cannabis use were inaccurate. We also did not have information pertaining to the types of cannabis products used (eg, cannabis oil concentrates and other formulations). As in other longitudinal MRI studies, there is uncertainty with regard to the exact neurobiological mechanisms associated with MRI-assessed cortical thinning. Research suggests that MRI-assessed, age-related cortical thinning may reflect increased myelination of lower cortical layers as opposed to synaptic pruning and/or neuronal cell loss.^57^ Natu et al^57^ found good correspondence between MRI-assessed cortical thickness and histologic measurements of cortical thickness among young adults. This latter finding is critical given that we detected cannabis-related differences in cortical thickness at age 19 years and not at 14 years, suggesting that our MRI-assessed cortical thickness findings are associated with reduced cortical gray matter rather than increased myelination. The present study focused on cortical thickness development and did not examine potential cannabis-related outcomes within subcortical structures. Future studies may benefit from conducting similar analyses on subcortical regions, particularly those rich in CB1 receptors. Most important, given the observational nature of the present study, it is possible that the apparent association between cortical thinning and cannabis use reflects preexisting trajectories of brain maturation that were not caused by cannabis use. We cannot rule out the possibility that preexisting cognitive and/or behavioral differences are associated with neurodevelopmental trajectories from adolescence to early adulthood and that cannabis use is not causally related to cerebral cortical thickness development. Although such an alternative explanation is possible, several observations from the present study are worth reiterating. First, there was a dose-dependent association at 5-year follow-up between lifetime cannabis use and cortical thickness. Second, there were no significant associations between baseline cortical thickness and lifetime cannabis use at 5-year follow-up. Given evidence of first-order monotonic thinning for much of the cerebral cortex during childhood and adolescence,^8,33^ it would seem unlikely that differing maturational trajectories, if present, would not have been detectable at baseline. Third, the spatial pattern of cannabis-related thinning was significantly associated with a PET-derived map of CB1 receptor availability.

## Conclusions

To our knowledge, the present investigation represents the largest longitudinal neuroimaging study of adolescent cannabis use to date. We report evidence of an association between adolescent cannabis use and altered cortical thickness development in a longitudinal sample of youths. The spatial pattern of cannabis-related thinning was associated with a PET-derived map of CB1 receptor availability as well as a map of age-related thickness change. The findings underscore the importance of further longitudinal studies of adolescent cannabis use, particularly given increasing trends in the legalization of recreational cannabis use.

## Notes

Supplement. **eAppendix 1.** MRI Acquisition and Image Processing **eAppendix 2.** Random Field Theory Correction **eAppendix 3.** Demographic Measures **eAppendix 4.** Between-Group Analyses **eAppendix 5.** Cannabis-Related Thinning and Impulsiveness **eAppendix 6.** Cannabis Use, Cannabis-Related Cortical Thinning and Neurocognition **eAppendix 7.** Cannabis Use, Cannabis-Related Cortical Thinning and Psychopathology **eTable 1.** Demographic Summary at Varying Cannabis Use Levels **eTable 2.** Demographics of Excluded Participants **eTable 3.** ESPAD Baseline Summary **eTable 4.** ESPAD Follow-Up Summary **eTable 5.** AUDIT Baseline Summary **eTable 6.** AUDIT Follow-Up Summary **eFigure 1.** Histogram of Lifetime Cannabis Use at 5-Year Follow-Up **eFigure 2.** Baseline Local Cortical Thickness and Lifetime Cannabis Use at Follow-Up **eFigure 3.** Cortical Thickness and Change in Lifetime Cannabis Use **eFigure 4.** Results of Between-Group Cross-Sectional Analysis **eFigure 5.** Results of Between-Group Longitudinal Analysis **eFigure 6.** Controlling for Lifetime Tobacco Use
eReferences
