# Supplementary material for: Association of Cannabis Use During Adolescence With Neurodevelopment
Source: JAMA Psychiatry. 2021 Jun 16;78(9):1–11. doi: 10.1001/jamapsychiatry.2021.1258 (PMC8209561; doi:10.1001/jamapsychiatry.2021.1258)
Supplement: Supplement. — eAppendix 1. MRI Acquisition and Image Processing eAppendix 2. Random Field Theory Correction eAppendix 3. Demographic Measures eAppendix 4. Between-Group Analyses eAppendix 5. Cannabis-Related Thinning and Impulsiveness eAppendix 6. Cannabis Use, Cannabis-Related Cortical Thinning and Neurocognition eAppendix 7. Cannabis Use, Cannabis-Related Cortical Thinning and Psychopathology eTable 1. Demographic Summary at Varying Cannabis Use Levels eTable 2. Demographics of Excluded Participants eTable 3. ESPAD Baseline Summary eTable 4. ESPAD Follow-Up Summary eTable 5. AUDIT Baseline Summary eTable 6. AUDIT Follow-Up Summary eFigure 1. Histogram of Lifetime Cannabis Use at 5-Year Follow-Up eFigure 2. Baseline Local Cortical Thickness and Lifetime Cannabis Use at Follow-Up eFigure 3. Cortical Thickness and Change in Lifetime Cannabis Use eFigure 4. Results of Between-Group Cross-Sectional Analysis eFigure 5. Results of Between-Group Longitudinal Analysis eFigure 6. Controlling for Lifetime Tobacco Use eReferences [file jamapsychiatry-e211258-s001.pdf]

Supplementary Online Content

Albaugh MD, Ottino-Gonzalez J, Sidwell A, et al; IMAGEN Consortium. Association of cannabis use during adolescence with neurodevelopment. *JAMA Psychiatry.* Published online June 16, 2021.
doi:10.1001/jamapsychiatry.2021.1258

eAppendix 1. MRI Acquisition and Image Processing

eAppendix 2. Random Field Theory Correction

eAppendix 3. Demographic Measures

eAppendix 4. Between-Group Analyses

eAppendix 5. Cannabis-Related Thinning and Impulsiveness

eAppendix 6. Cannabis Use, Cannabis-Related Cortical Thinning and Neurocognition

eAppendix 7. Cannabis Use, Cannabis-Related Cortical Thinning and Psychopathology

eTable 1. Demographic Summary at Varying Cannabis Use Levels

eTable 2. Demographics of Excluded Participants

eTable 3. ESPAD Baseline Summary

eTable 4. ESPAD Follow-Up Summary

eTable 5. AUDIT Baseline Summary

eTable 6. AUDIT Follow-Up Summary

eFigure 1. Histogram of Lifetime Cannabis Use at 5-year Follow-Up

eFigure 2. Baseline Local Cortical Thickness and Lifetime Cannabis Use at Follow-Up

eFigure 3. Cortical Thickness and Change in Lifetime Cannabis Use

eFigure 4. Results of Between-Group Cross-Sectional Analysis

eFigure 5. Results of Between-Group Longitudinal Analysis

eFigure 6. Controlling for Lifetime Tobacco Use

eReferences

This supplementary material has been provided by the authors to give readers additional information about their work.

© 2021 Albaugh MD et al. *JAMA Psychiatry*.

eAppendix 1. MRI Acquisition and Image Processing

MRI scanning was conducted at the eight IMAGEN assessment sites using 3T whole body MRI systems.[1] Image-acquisition utilized parameters that were compatible with all scanners in order to ensure comparability of data across the different scanners. Details surrounding image acquisition protocols and quality checks have been described elsewhere, including extensive standardization across MRI scanner.[1]

The CIVET pipeline was used for extraction of cortical surfaces and estimation of local cortical thickness. The following steps were performed as part of this processing pipeline.[2] Native MR images were linearly registered to a standardized MNI-Talairach space based on the ICBM152 dataset.[3-6] Intensity non-uniformity artifacts were corrected for using the N3 algorithm.[7] Brain extraction was implemented using FSL's Brain Extraction Tool (BET).[8] Classification of white matter (WM), gray matter (GM), and cerebrospinal fluid (CSF) was carried out using the INSECT algorithm.[9,10] The CIVET pipeline includes the CLASP algorithm used to generate high-resolution hemispheric surfaces with 40,962 vertices per hemisphere.[11-14] Hemispheric surfaces were generated for both the WM/GM interface, as well as the GM/CSF interface. In order to establish correspondence of vertices between subjects, the surfaces for each hemisphere were non-linearly registered to an average surface created from the ICBM152 dataset.[12,15] A reverse linear transformation was performed on each subject's images, allowing for cortical thickness estimations to be made at each cortical point in the MR image's native space.[16] To increase the signal-to-noise ratio, each subject's cortical thickness map was blurred using a 20-millimeter full width at half maximum surface-based diffusion smoothing kernel.[15] This kernel size closely approximates previously recommended values, affording optimal sensitivity for cortical thickness analysis.[17] Following from previous work by members of

© 2021 Albaugh MD et al. *JAMA Psychiatry*.

our group, quality control of CIVET output was performed with regard to (a) registration, (b) surface extraction, and (c) gray-white surface-surface intersections.[18] Absolute cortical thickness measures were also reviewed as extreme values can provide cues of poor surface recognition.

© 2021 Albaugh MD et al. *JAMA Psychiatry*.

eAppendix 2. Random Field Theory Correction

In order to identify significant clusters, an initial height threshold of $p \leq .001$ was implemented at the vertex level, and a corrected family-wise error ($p \leq .05$) was subsequently applied. Vertex-level RFT thresholding was implemented using the vertex-wise RFT critical *t*-value which was calculated from the expected Euler characteristic and number of resolution elements, or resels.[19]

© 2021 Albaugh MD et al. *JAMA Psychiatry*.

eAppendix 3. Demographic Measures

The socioeconomic status (SES) score was derived by summing the following variables: Mother's Education Score, Father's Education Score, Family Stress Unemployment Score, Financial Difficulties Score, Home Inadequacy Score, Neighborhood Score, Financial Crisis Score, Mother Employed Score, and Father Employed Score.[20] Participants completed the Perceptual Reasoning, Matrix Reasoning, Similarities and Vocabulary subscales from the Wechsler intelligence scale for children WISC-IV),[21] and Verbal Comprehension (VCIQ) and Perceptual Reasoning (PRIQ) indices were generated.

© 2021 Albaugh MD et al. *JAMA Psychiatry*.

eAppendix 4. Between-Group Analyses

It bears noting that nearly identical results were obtained when primary analyses in present study were rerun using a between-group design (i.e., participants reporting moderate-to-heavy lifetime cannabis use at 5-year follow-up versus those who remained cannabis-naïve). In particular, moderate-to-heavy lifetime cannabis users were defined as participants endorsing 10-19 uses or more (i.e., ≥ 4) on the lifetime cannabis use item at 5-year-follow-up. At 5-year follow-up, participants reporting moderate-to-heavy lifetime cannabis use (n = 161) exhibited reduced cortical thickness in a number of prefrontal areas relative to those who remained cannabis-naïve (n = 430). No significant between-group differences were observed with regard to baseline cortical thickness. Linear mixed-effects model analysis (591 subjects; 1182 MRIs) revealed accelerated age-related cortical thinning among participants who transitioned to moderate-to-heavy lifetime cannabis use relative to those who remained cannabis-naïve. See eFigures 4 and 5, below.

© 2021 Albaugh MD et al. *JAMA Psychiatry*.

eAppendix 5. Cannabis-Related Thinning and Impulsiveness

Of the 799 participants in the present study, 697 (87.2%) had available Barratt Impulsivity Scale (BIS) data at 5-year follow-up (this measure was not administered at baseline). Average thickness (at baseline and follow-up) was calculated for each significant cluster in the LMM analysis examining the influence of cannabis use on age-related cortical thinning. For all participants, symmetrized percent change (SPC) (i.e., change in cortical thickness, in millimeters per year, with respect to the mean cortical thickness across both time points) was calculated for each significant cluster (i.e., right dorsomedial prefrontal, left dorsal prefrontal, and left inferior parietal clusters). Given that three BIS scales were tested in each of the three cortical regions, a corrected *p* value of 0.006 was adopted (0.05/9). Critically, cannabis-related cortical thinning in the right dorsal prefrontal cortex accounted for unique variance in attentional impulsiveness at 5-year follow-up while controlling for sex, site, baseline age, baseline brain volume, baseline pubertal development, IQPR, and IQVC (b = -.119, p = .003).

We reran our analysis examining the extent to which symmetrized percent change (SPC) in the right dorsomedial prefrontal cluster was uniquely associated with attentional impulsivity at follow-up while controlling for both parent and youth reports of ADHD symptomatology at baseline. Notably, the association was still significant. Results from the GLM are displayed below.

© 2021 Albaugh MD et al. *JAMA Psychiatry*.

Tests of Between-Subjects Effects

Dependent Variable: BIS_SUM_Attention

Source	Type III Sum of Squares	df	Mean Square	F	Sig.
Corrected Model	1526.954[a]	16	95.435	10.785	.000
Intercept	69.235	1	69.235	7.824	.005
Sex	.037	1	.037	.004	.949
Site	191.145	7	27.306	3.086	.003
Baseline TBV	8.338	1	8.338	.942	.332
Baseline Pubertal Development	40.551	1	40.551	4.583	.033
Baseline Age	.038	1	.038	.004	.948
IQPR	13.136	1	13.136	1.484	.223
IQVC	4.699	1	4.699	.531	.466
SDQ Hyperactive/Impulsive Score	806.318	1	806.318	91.124	.000
DAWBA Symptom Score	26.863	1	26.863	3.036	.082
Right dmPFC (spc)	43.113	1	43.113	4.872	.028
Error	5990.466	677	8.849		
Total	173075.000	694			
Corrected Total	7517.419	693			

a. R Squared = .203 (Adjusted R Squared = .184)

© 2021 Albaugh MD et al. *JAMA Psychiatry*.

eAppendix 6. Cannabis Use, Cannabis-Related Cortical Thinning and Neurocognition

In a series of post hoc analyses, we examined associations between change in cannabis use (from baseline to follow-up) and neurocognitive measures at baseline and follow-up (assessed using the Cambridge Neuropsychological Test Automated Battery, or CANTAB). We conducted a series of partial correlations between neurocognitive measures and change in cannabis use (from baseline to follow-up), controlling for sex, age at time of testing, and SES. Importantly, there were no significant associations between baseline measures and change in cannabis use. At follow-up, two measures were only nominally associated with change in cannabis use: overall proportion of bets ($p = 0.02$) and risk taking ($p = 0.04$) on the Cambridge Gambling Task. These associations did not survive correction for the number of tests conducted.

We also tested for associations between cortical thickness change in prefrontal regions exhibiting cannabis-related thinning (i.e., significant clusters from the LMM analysis) and neurocognitive measures at baseline and follow-up (assessed using the Cambridge Neuropsychological Test Automated Battery, or CANTAB). Sex, age at baseline, site, baseline total brain volume, baseline pubertal stage, and baseline IQ were accounted for across analyses. Across all tests, only nominally significant associations were observed between Total Omissions Negative on the Affective Go-NoGo (AGN) task at follow-up and cannabis-related thinning in left and right dorsomedial PFC ($p = 0.02$ and $p = 0.04$, respectively). These associations would not survive formal correction for multiple comparisons. Thus, it would seem unlikely that our observed cannabis-related brain effects are driven by pre-existing neurocognitive factors.

© 2021 Albaugh MD et al. *JAMA Psychiatry*.

eAppendix 7. Cannabis Use, Cannabis-Related Cortical Thinning and Psychopathology

In a series of post hoc partial correlations, we examined potential associations between change in cannabis use (baseline to follow-up), cannabis-related cortical thinning, and psychopathology measures (baseline and follow-up). Sex, age at baseline, site, baseline total brain volume, baseline pubertal stage, baseline SES, and baseline verbal and performance IQ were controlled for across analyses. Change in cannabis use was associated with conduct problems at both time points, total emotional and behavior problems at follow-up, and psychotic symptoms (only assessed at follow-up).

© 2021 Albaugh MD et al. *JAMA Psychiatry*.

Partial Correlations

		Change in Cannabis Use	L dmPFC SPC	R dmPFC SPC
Change in Cannabis Use	Correlation	1.000	-.105	-.117
	Significance (2-tailed)	.	.003	.001
	df	0	783	783
L dmPFC SPC	Correlation	-.105	1.000	.651
	Significance (2-tailed)	.003	.	.000
	df	783	0	783
R dmPFC SPC	Correlation	-.117	.651	1.000
	Significance (2-tailed)	.001	.000	.
	df	783	783	0
SDQ Emotion Problems (baseline)	Correlation	-.025	-.047	-.003
	Significance (2-tailed)	.486	.184	.930
	df	783	783	783
SDQ Emotion Problems (follow-up)	Correlation	.030	.004	.009
	Significance (2-tailed)	.407	.910	.792
	df	783	783	783
SDQ Conduct Problems (baseline)	Correlation	.140	-.019	-.033
	Significance (2-tailed)	.000	.592	.358
	df	783	783	783
SDQ Conduct Problems (follow-up)	Correlation	.131	.012	.003
	Significance (2-tailed)	.000	.731	.937
	df	783	783	783
SDQ Total Emotional and Behavioral Difficulties (baseline)	Correlation	.045	-.036	-.049
	Significance (2-tailed)	.211	.311	.171
	df	783	783	783
SDQ Total Emotional and Behavioral Difficulties (follow-up)	Correlation	.098	.009	-.007
	Significance (2-tailed)	.006	.795	.834
	df	783	783	783
Parent DAWBA ADHD Symptom Severity (baseline)	Correlation	.039	.076	.011
	Significance (2-tailed)	.281	.035	.760
	df	780	780	780
CAPE Total Score (follow-up)	Correlation	.170	.002	-.019
	Significance (2-tailed)	.000	.951	.605
	df	752	752	752

Across analyses, there was little evidence of significant associations between psychopathology and cannabis-related cortical thinning. Only parent-reported ADHD psychopathology was nominally associated with cannabis-related thinning in the left dorsomedial prefrontal region ($p = 0.035$). Critically, the association between cannabis-related prefrontal cortical thinning and change in cannabis use (from baseline to follow-

© 2021 Albaugh MD et al. *JAMA Psychiatry*.

up) remained significant even after partialling out all of the listed psychopathology scores (including parent-reported ADHD symptomatology).

We also tested for associations between cortical thickness change in prefrontal regions exhibiting cannabis-related thinning (i.e., significant clusters from the LMM analysis) and change in psychopathology scores (from baseline and follow-up) (assessed using the Strength and Difficulties Questionnaire, or SDQ). Sex, age at baseline, site, baseline total brain volume, baseline pubertal stage, baseline SES, and baseline IQ were accounted for across analyses. Across all tests, no significant associations were found.

Partial Correlations

			L dmPFC SPC	R dmPFC SPC
	Change in SDQ Conduct Problems	Correlation	.031	.034
		Significance (2-tailed)	.384	.339
		df	783	783
	Change in SDQ Emotion Problems	Correlation	.044	.008
		Significance (2-tailed)	.223	.824
		df	783	783
	Change in SDQ Total Emotional and Behavioral Difficulties	Correlation	.042	.036
		Significance (2-tailed)	.241	.317
		df	783	783
	Change in SDQ Hyperactive/Inattentive	Correlation	.054	.047
		Significance (2-tailed)	.130	.190
		df	783	783

© 2021 Albaugh MD et al. *JAMA Psychiatry*.

eTable 1. Demographic Summary at Varying Cannabis Use Levels

	0 Lifetime Uses at Follow-Up	1 to 9 Lifetime Uses at Follow-Up	10 to >40 Lifetime Uses at Follow-Up
Age at baseline (in years) (Mean ± SD)	14.46 ± 0.41	14.39 ± 0.38	14.43 ± 0.40
Age at follow-up (in years) (Mean ± SD)	18.92 ± 0.66	19.00 ± 0.70	19.08 ± 0.76
Sex	284 Females, 146 Males	112 Females, 96 Males	54 Females, 107 Males
Baseline SES (Mean ± SD)	18.06 ± 3.61	18.63 ± 3.63	18.15 ± 4.05
Baseline Verbal IQ (Mean ± SD)	111.94 ± 13.53	112.85 ± 12.41	114.17 ± 12.39
Baseline Performance IQ (Mean ± SD)	110.25 ± 13.84	109.41 ± 13.21	107.86 ± 13.55
Baseline DAWBA ADHD Symptom Severity (Mean ± SD)	2.89 ± 5.03	3.71 ± 5.77	3.79 ± 5.34
Baseline SDQ Total Emotional and Behavioral Problems (Mean ± SD)	10.25 ± 4.84	9.99 ± 4.12	10.04 ± 4.28
Follow-up SDQ Total Emotional and Behavioral Problems (Mean ± SD)	9.58 ± 4.89	9.82 ± 4.60	10.01 ± 4.74

A Chi-square test and one-way ANOVAs revealed that groups significantly differed only with regard to sex (X^2 = 51.01, $p < 0.05$) and age at follow-up (F = 3.16, $p = 0.043$).

© 2021 Albaugh MD et al. *JAMA Psychiatry*.

eTable 2. Demographics of Excluded Participants

	Sample (N = 799)	Excluded Participants
Age at baseline (in years) (Mean ± SD)	14.43 ± 0.40	14.55 ± 0.44 (N = 1258)**
Sex	56.3% F (450), 43.7% M (349)	52.2% F (662), 47.8% M (607) (N = 1269)
Baseline SES (Mean ± SD)	18.23 ± 3.71	17.48 ± 4.18 (N = 1265)**
Baseline Verbal IQ (Mean ± SD)	112.63 ± 13.04	108.39 ± 18.08 (N = 1116)**
Baseline Performance IQ (Mean ± SD)	109.55 ± 13.64	105.96 ± 15.22 (N = 1116)**

** = $p < 0.05$ for corresponding t-test or chi-square

When comparing participants included in the present study with participants excluded for either baseline cannabis use or incomplete imaging data, participants in the present study were found to have significantly higher SES and IQ scores. A chi-square test revealed that included and excluded participants did not significantly differ with regard to sex.

© 2021 Albaugh MD et al. *JAMA Psychiatry*.

eTable 3. ESPAD Baseline Summary

ESPAD Baseline					
	N	Minimum	Maximum	Mean	Std. Deviation
Lifetime amphetamines	799	.00	.00	.0000	.00000
Lifetime anabolic steroids	799	.00	1.00	.0025	.05000
Lifetime cocaine	799	.00	2.00	.0025	.07075
Lifetime crack	799	.00	1.00	.0013	.03538
Lifetime GHB	799	.00	.00	.0000	.00000
Lifetime glue	799	.00	5.00	.0338	.25549
Lifetime heroin	799	.00	1.00	.0013	.03538
Lifetime ketamine	799	.00	.00	.0000	.00000
Lifetime LSD	799	.00	2.00	.0038	.07907
Lifetime MDMA	799	.00	2.00	.0038	.07907
Lifetime mushrooms	799	.00	1.00	.0013	.03538
Lifetime narcotics	799	.00	2.00	.0100	.12222
Lifetime tranquilizers	799	.00	6.00	.0213	.25683
Lifetime cannabis/hash	799	.00	.00	.0000	.00000
Lifetime nicotine	799	.00	6.00	.4418	1.1531

*Scoring on ESPAD items is as follows: 0: never, 1: 1–2 times, 2: 3–5 times, 3: 6 –9 times, 4: 10 –19 times, 5: 20 –39 times, and 6: 40 or more times.

© 2021 Albaugh MD et al. *JAMA Psychiatry*.

eTable 4. ESPAD Follow-Up Summary

ESPAD 5-year Follow-up					
	N	Minimum	Maximum	Mean	Std. Deviation
Lifetime amphetamines	799	.00	6.00	.1126	.64098
Lifetime anabolic steroids	799	.00	.00	.0000	.00000
Lifetime cocaine	799	.00	5.00	.0876	.47211
Lifetime crack	799	.00	1.00	.0013	.03538
Lifetime GHB	799	.00	1.00	.0038	.06120
Lifetime glue	799	.00	6.00	.0551	.39984
Lifetime heroin	799	.00	.00	.0000	.00000
Lifetime ketamine	799	.00	6.00	.0463	.38663
Lifetime LSD	799	.00	2.00	.0238	.16809
Lifetime MDMA	799	.00	6.00	.2365	.89155
Lifetime mushrooms	799	.00	4.00	.0363	.23995
Lifetime narcotics	799	.00	6.00	.0238	.29733
Lifetime tranquilizers	799	.00	2.00	.0088	.11708
Lifetime cannabis/hash	799	.00	6.00	1.5006	2.09240
Lifetime nicotine	799	.00	6.00	2.5031	2.45198

*Scoring on ESPAD items is as follows: 0: never, 1: 1–2 times, 2: 3–5 times, 3: 6 –9 times, 4: 10 –19 times, 5: 20 –39 times, and 6: 40 or more times.

© 2021 Albaugh MD et al. *JAMA Psychiatry*.

eTable 5. **AUDIT Baseline Summary**

AUDIT Alcohol Consumption Baseline					
	N	Minimum	Maximum	Mean	Std. Deviation
AUDIT Consump	799	.0	8.0	.877	1.2982

© 2021 Albaugh MD et al. *JAMA Psychiatry*.

eTable 6. **AUDIT Follow-Up Summary**

AUDIT Alcohol Consumption 5-year Follow-up					
	N	Minimum	Maximum	Mean	Std. Deviation
AUDIT Consump	799	.0	11.0	4.124	2.4769

© 2021 Albaugh MD et al. *JAMA Psychiatry*.

eFigure 1. Histogram of Lifetime Cannabis Use at 5-Year Follow-Up

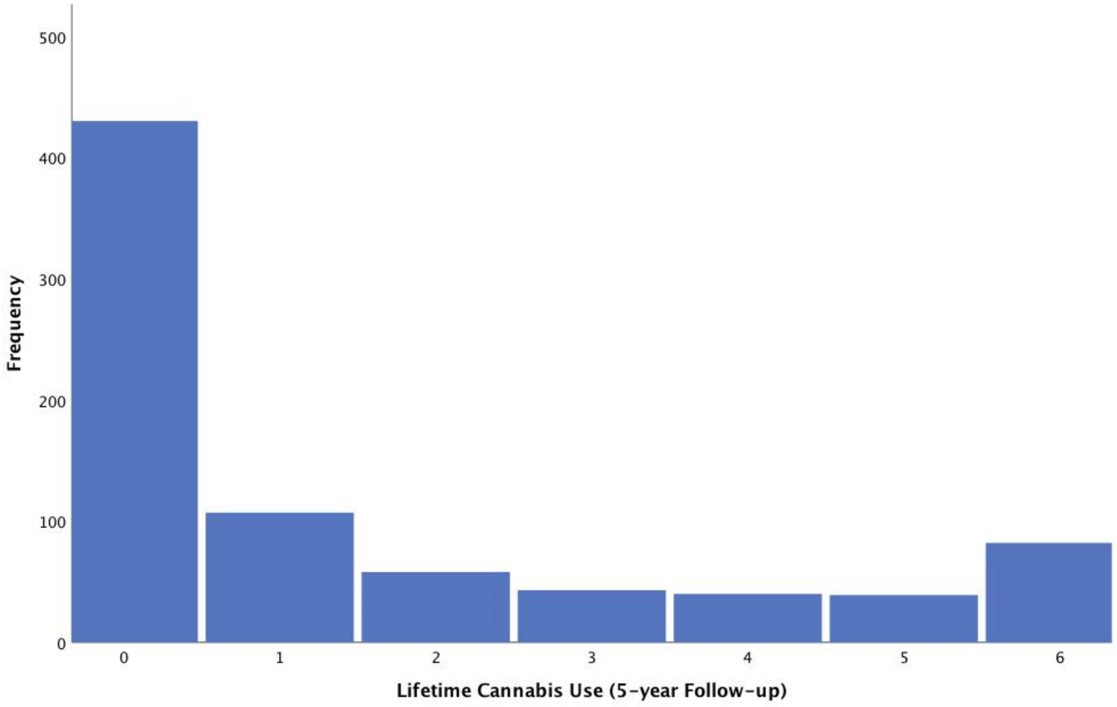

Histogram of lifetime cannabis use at 5-year follow-up (0: never, 1: 1–2 times, 2: 3–5 times, 3: 6 –9 times, 4: 10 –19 times, 5: 20 –39 times, and 6: 40 or more times).

© 2021 Albaugh MD et al. *JAMA Psychiatry*.

eFigure 2. **Baseline Local Cortical Thickness and Lifetime Cannabis Use at Follow-Up**

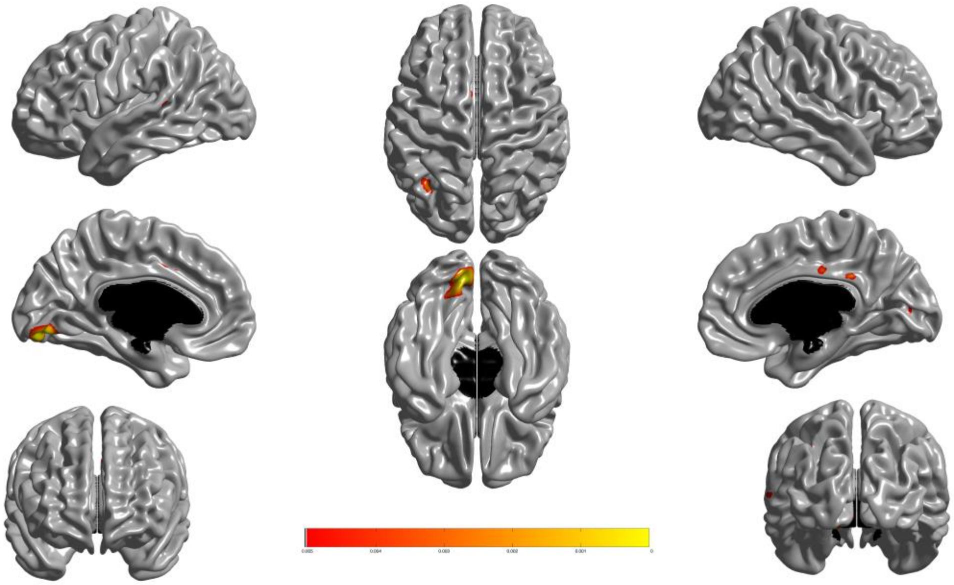

Brain areas where baseline local cortical thickness is negatively associated with dimensional measure of lifetime cannabis use at 5-year follow-up (n = 799). Figure is shown at $p \leq 0.005$, uncorrected. Controlled for age, total brain volume, sex, handedness, AUDIT Alcohol Consumption score, and site. No regions passed threshold for positive associations.

© 2021 Albaugh MD et al. *JAMA Psychiatry*.

eFigure 3. **Cortical Thickness and Change in Lifetime Cannabis Use**

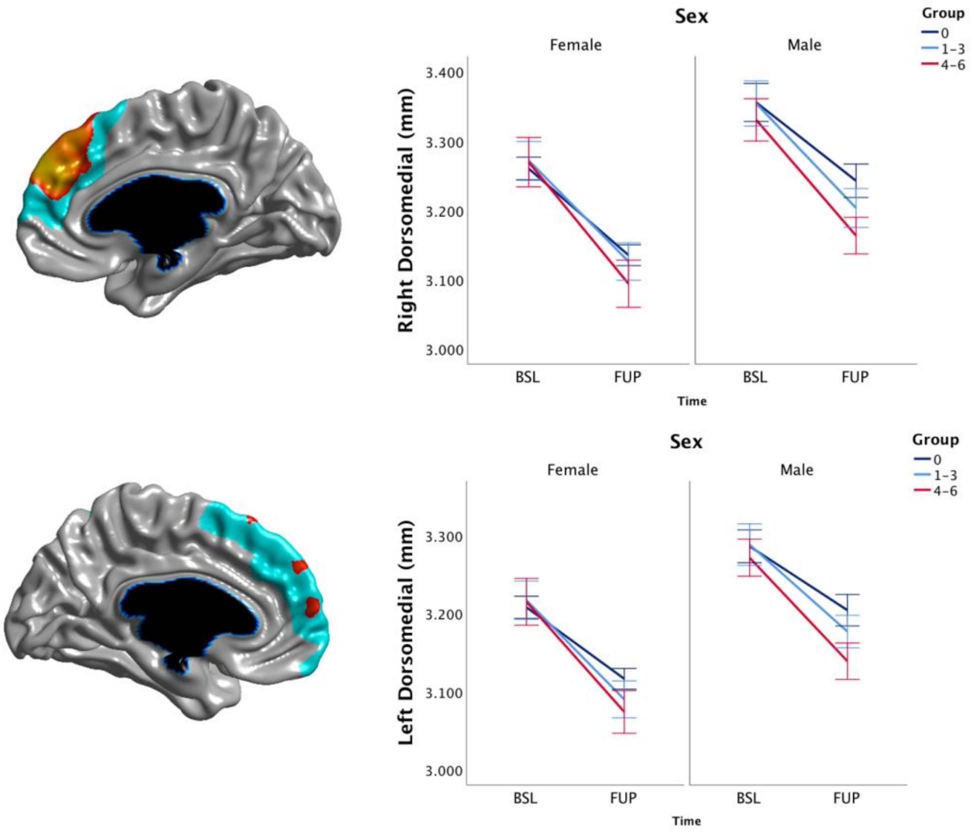

Line graphs depicting the relationship between mean cortical thickness (unadjusted) in prefrontal clusters from linear mixed-effects model analysis (from baseline to 5-year follow-up) and change in lifetime cannabis use, with males and females plotted separately (*n* = 799; 1598 MRIs). For illustration purposes, lifetime cannabis use is grouped into ESPAD scores of 0, 1-3, and 4-6 (0 uses, 1-9 uses, and 10+ uses, respectively). Group 0: 284 females/146 males, Group 1-3: 112 females/96 males, Group 4-6: 54 females/107 males. BSL = baseline, and FUP = follow-up. Error bars represent 95% confidence intervals.

© 2021 Albaugh MD et al. *JAMA Psychiatry*.

eFigure 4. Results of Between-Group Cross-Sectional Analysis

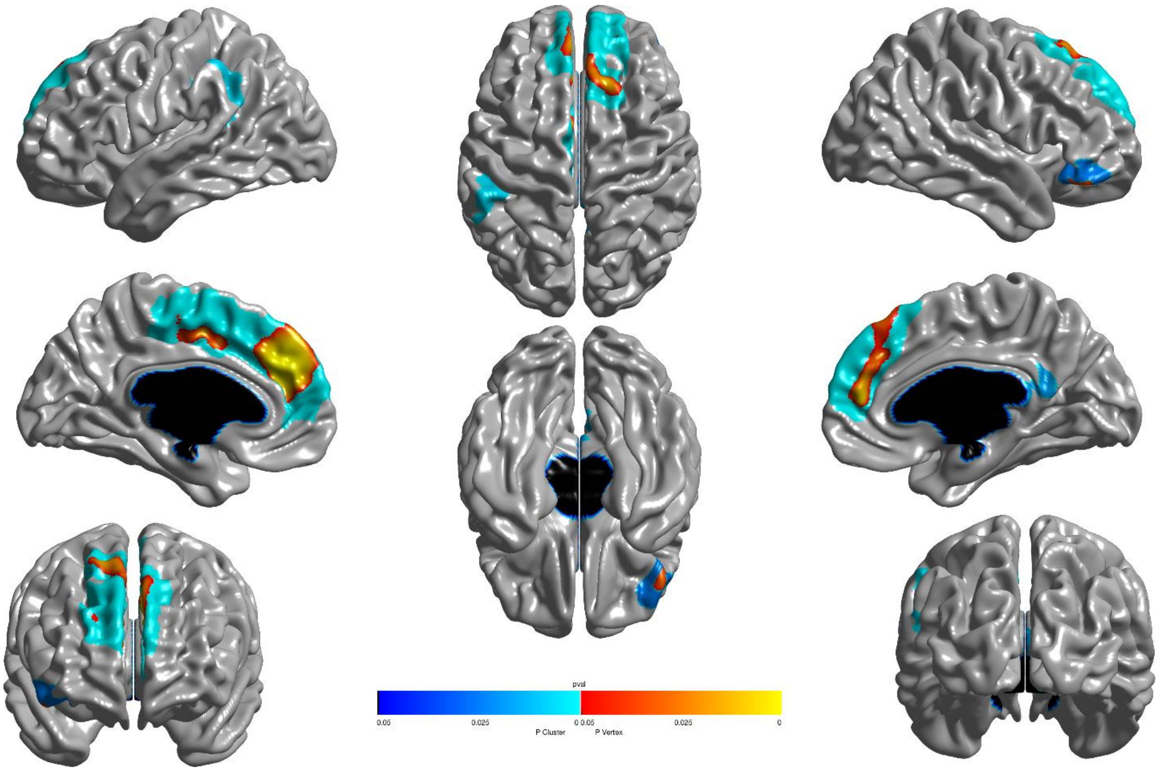

Brain areas where, at 5-year follow-up, local cortical thickness was significantly reduced in participants reporting moderate-to-heavy lifetime cannabis use (n = 161) relative to participants who remained cannabis-naïve (n = 430). Random field theory (RFT) was used to correct for multiple comparisons over the entire cortical mantle. Figure is shown at $p \leq 0.05$, RFT corrected. Blue areas are significant at the cluster level and red color corresponds to areas significant at the vertex level. Controlled for age, total brain volume, sex, handedness, AUDIT Alcohol Consumption, and site.

© 2021 Albaugh MD et al. *JAMA Psychiatry*.

eFigure 5. **Results of Between-Group Longitudinal Analysis**

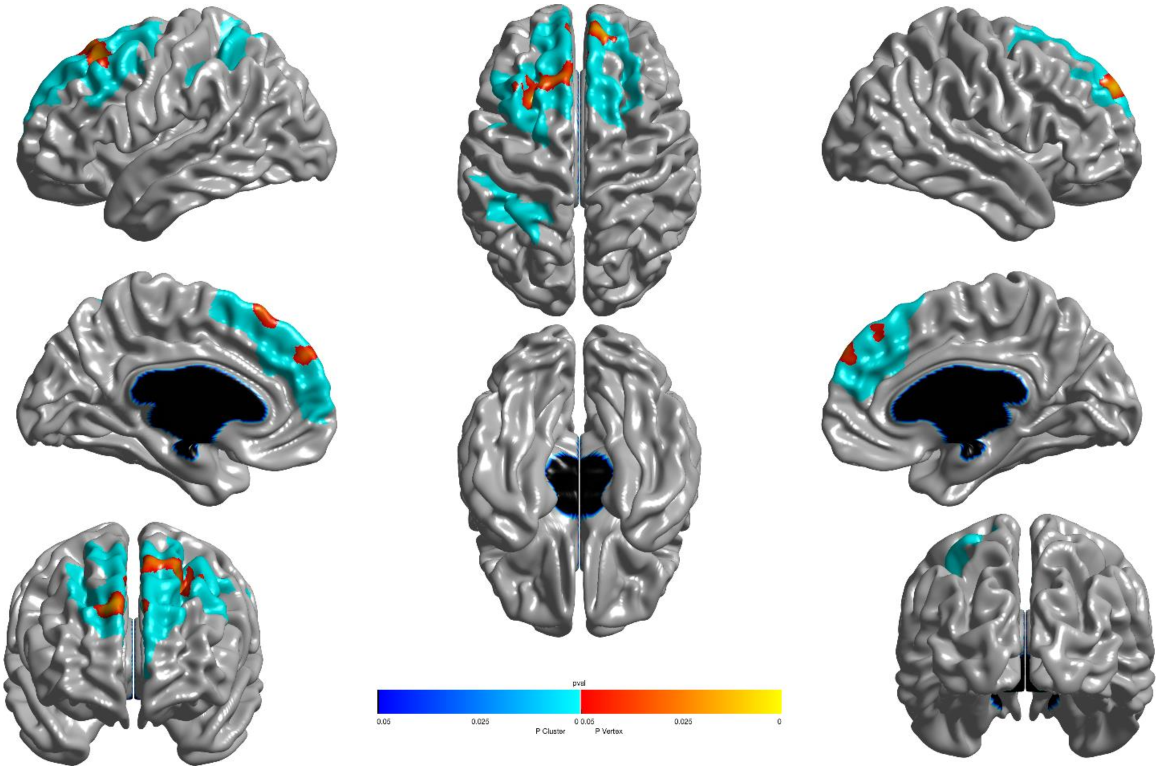

Brain areas where local cortical thickness is associated with the Group*Time interaction in a linear mixed-effects model analysis, controlling for the main effects of time point, group status (i.e., participants who transitioned to moderate-to-heavy lifetime cannabis use versus controls who remained cannabis-naïve), total brain volume, sex, handedness, AUDIT Alcohol Consumption score, and site (n = 591; 1182 MRI scans). Figure is shown at $p \leq 0.05$ with a whole-brain random field theory correction. Blue shades correspond to areas significant at the cluster level and orange shades to areas significant at the vertex level.

© 2021 Albaugh MD et al. *JAMA Psychiatry*.

eFigure 6. **Controlling for Lifetime Tobacco Use**

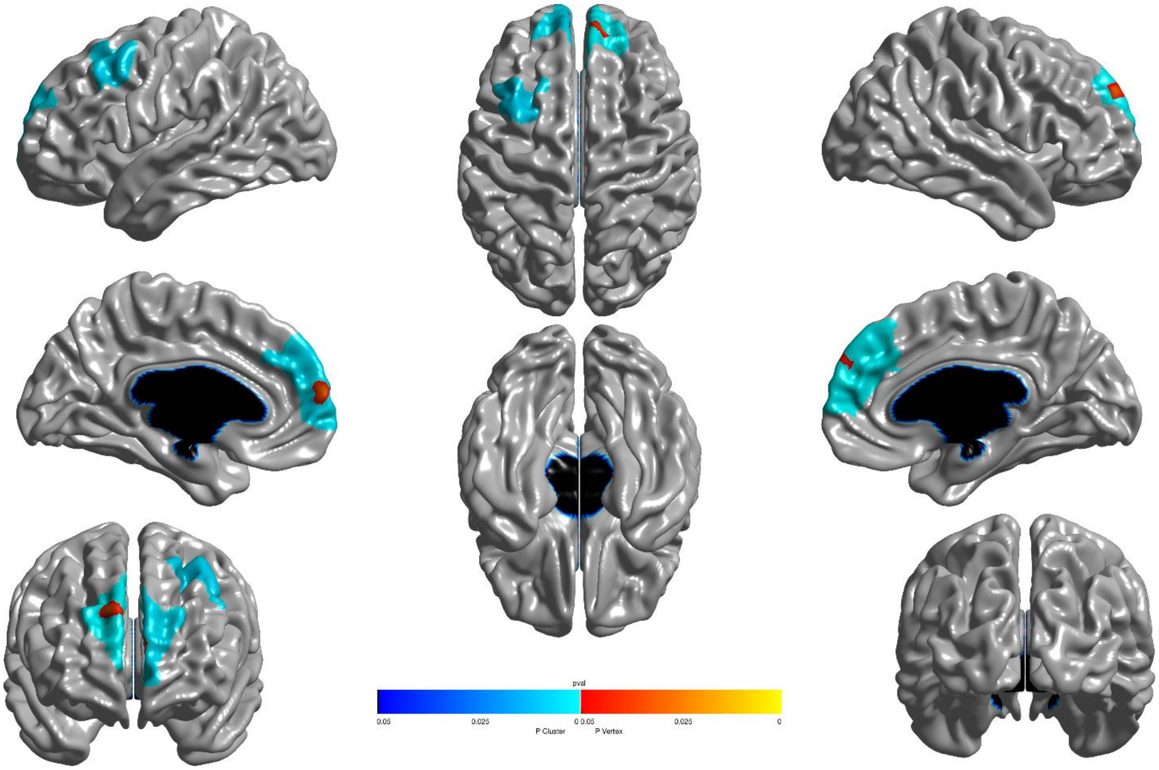

Brain areas where local cortical thickness is associated with the Time*Cannabis Use interaction in a linear mixed-effects model analysis, controlling for the main effects of time point, lifetime cannabis use, total brain volume, sex, handedness, AUDIT Alcohol Consumption score, lifetime tobacco use, and site (n = 799; 1598 MRI scans). Figure is shown at p ≤ 0.05 with a whole-brain random field theory correction. Blue shades correspond to areas significant at the cluster level and orange shades to areas significant at the vertex level.

© 2021 Albaugh MD et al. *JAMA Psychiatry*.

eReferences

1. Schumann G, Loth E, Banaschewski T, et al. The IMAGEN study: reinforcement-related behaviour in normal brain function and psychopathology. *Mol Psychiatry.* 2010;15(12):1128-1139.
2. Lepage CY, Lewis LB, Jeun S, et al. Human MR Evaluation of Cortical Thickness Using CIVET v2.1. Organization for Human Brain Mapping; 2017; Vancouver.
3. Collins DL, Neelin P, Peters TM, Evans AC. Automatic 3D intersubject registration of MR volumetric data in standardized Talairach space. *J Comput Assist Tomogr.* 1994;18(2):192-205.
4. Mazziotta JC, Toga AW, Evans A, Fox P, Lancaster J. A probabilistic atlas of the human brain: theory and rationale for its development. The International Consortium for Brain Mapping (ICBM). *Neuroimage.* 1995;2(2):89-101.
5. Talairach J, Tournoux P. *Co-planar stereotaxic atlas of the human brain : 3-dimensional proportional system : an approach to cerebral imaging.* Stuttgart ; New York: Georg Thieme; 1988.
6. Fonov VS, Evans AC, McKinstry RC, Almli CR, Collins DL. Unbiased nonlinear average age-appropriate brain templates from birth to adulthood. Orgnization for Human Brain Mapping; July 2009, 2009; San Francisco, CA.
7. Sled JG, Zijdenbos AP, Evans AC. A nonparametric method for automatic correction of intensity nonuniformity in MRI data. *IEEE Trans Med Imaging.* 1998;17(1):87-97.
8. Smith SM. Fast robust automated brain extraction. *Human brain mapping.* 2002;17(3):143-155.
9. Zijdenbos AP, Forghani R, Evans AC. Automatic "pipeline" analysis of 3-D MRI data for clinical trials: application to multiple sclerosis. *IEEE Trans Med Imaging.* 2002;21(10):1280-1291.
10. Tohka J, Zijdenbos A, Evans A. Fast and robust parameter estimation for statistical partial volume models in brain MRI. *Neuroimage.* 2004;23(1):84-97.
11. Kim J, Singh V, MacDonald D, Lee J, Kim S, Evans A. Automated 3D extraction and evaluation of the outer cortical surface using a Laplacian map and partial volume effect classification. *Neuroimage.* 2005;27:210-221.
12. Lyttelton O, Boucher M, Robbins S, Evans A. An unbiased iterative group registration template for cortical surface analysis. *Neuroimage.* 2007;34(4):1535-1544.
13. MacDonald D, Kabani N, Avis D, Evans A. Automated 3D extraction of inner and outer surfaces of cerebral cortex from MRI. *Neuroimage.* 2000;13:340-356.
14. Kabani N, Le Goualher G, MacDonald D, Evans AC. Measurement of cortical thickness using an automated 3-D algorithm: a validation study. *Neuroimage.* 2001;13(2):375-380.

© 2021 Albaugh MD et al. *JAMA Psychiatry*.

15. Boucher M, Whitesides S, Evans A. Depth potential function for folding pattern representation, registration and analysis. *Med Image Anal.* 2009;13(2):203-214.
16. Ad-Dab'bagh Y, Singh V, Robbins S, et al. Native space cortical thickness measurement and the absence of correlation to cerebral volume. 11th Annual Meeting of the Organization for Human Brain Mapping; 2005; Toronto.
17. Lerch JP, Evans AC. Cortical thickness analysis examined through power analysis and a population simulation. *Neuroimage.* 2005;24(1):163-173.
18. Ducharme S, Albaugh MD, Nguyen TV, et al. Trajectories of cortical thickness maturation in normal brain development--The importance of quality control procedures. *Neuroimage.* 2016;125:267-279.
19. Worsley KJ, Taylor JE, Tomaiuolo F, Lerch J. Unified univariate and multivariate random field theory. *Neuroimage.* 2004;23 Suppl 1:S189-195.
20. Whelan R, Watts R, Orr CA, et al. Neuropsychosocial profiles of current and future adolescent alcohol misusers. *Nature.* 2014;512(7513):185-189.
21. D W. *Wechsler intelligence scale for children.* New York: Psychological Corporation; 1949.

© 2021 Albaugh MD et al. *JAMA Psychiatry*.
